# ViewShedR: a new open-source tool for cumulative, subtractive and elevated line-of-sight analysis

**DOI:** 10.1098/rsos.221333

**Published:** 2023-06-28

**Authors:** Eitam Arnon, Assaf Uzan, Michal Handel, Shlomo Cain, Sivan Toledo, Orr Spiegel

**Affiliations:** ^1^ School of Zoology, Faculty of Life Sciences, Tel Aviv University, Tel Aviv 6997801, Israel; ^2^ Faculty of Architecture and Town Planning, Technion – Israel Institute of Technology, Haifa 3200003, Israel; ^3^ Blavatnik School of Computer Science, Tel Aviv University, Tel Aviv 6997801, Israel

**Keywords:** acoustic tracking, animal telemetry, animal tracking, intersective coverage, geospatial analysis, visibility analysis

## Abstract

Many environmental and ecological studies require line of sight (LOS) and/or viewshed analyses. While tools for performing these analyses from digital elevation models (DEMs) are widespread, they are either too restrictive, inaccessible or pricey and difficult to use. This methodological gap is potentially imperative for scholars using solutions like telemetry tracking systems or spatial ecology landscape mapping. Here we present ViewShedR—a free, open-source and intuitive graphical user-interface application for performing LOS calculations, including cumulative, subtractive (areas covered by towers A + B or by A but not by B, respectively), and elevated-target analyses. ViewShedR is implemented in the widely used R environment, thus facilitating usage and further modification by end-users. We provide two working examples for ViewShedR in the context of permanent animal-tracking systems requiring simultaneous tag-detection by multiple towers (receivers): first, the ATLAS system for terrestrial animals in the Harod Valley, Israel; and second, an acoustic telemetry array for marine animals in the Dry Tortugas, Florida. ViewShedR allowed effective tower deployment and finding partially detected tagged animals in the ATLAS system. Similarly, it allowed us to identify reception shadows cast by islands in the marine array. We hope ViewShedR will facilitate deployment of tower arrays for tracking, communication networks and other ecological applications.

## 1. Introduction

Many environmental and ecological studies require *line of sight* (LOS) analyses and/or viewshed analyses. A line of sight exists between two points, which we refer to as a *tower* and a *target* if the line segment connecting them lies above the terrain between them. A *viewshed* of a tower is the set of target points from which there is a line of sight to the tower [1].

The presence or absence of a LOS is essential for various novel technologies that require data retrieval or acquisition by communication or visual-observation towers. Interpretation of the topography and LOS calculations are being used in ecology, geography and environmental sciences, as well as in archaeology [2–5], and landscape architecture [6–9]. A prominent example for the importance of LOS comes from movement ecology.

Innovations in tracking technologies advance this emerging discipline by facilitating the measurement and description of animal movement and its consequences for ecological processes and conservation [10–14]. Several approaches to animal tracking rely on LOS between the animal-borne tags (targets in our terminology) and radio towers for localization of the tags or for retrieving data stored on board [15]. LOS is needed for classical radio triangulation methods [16] as well as for modern reverse-GPS methods, which are becoming increasingly popular in both terrestrial and aquatic studies [14,17–19]. While absorption, dispersion, reflections and other disturbances to the radio/acoustic signal may prevent a successful localization of a target through its triangulation or signals' time of arrival, the LOS provides the first-order approximation for signal detection ability. Thus, analytical tools for LOS calculations are instrumental for many studies, ecological and others, relying on similar technologies.

LOS calculations are based on comparing the elevation of a direct line segment between any two spatial positions and the elevation of the terrain under this line, as represented in a digital elevation model (DEM; sometimes referred as a digital *terrain* model, DTM). If calculated over distances longer than a few kilometres, LOS should ideally also account for earth curvature that can otherwise accumulate to considerable errors. Typically, viewshed analyses extend LOS calculations to a whole area around a tower by radiating the LOS calculation over chosen arch and range. Important extensions of basic analysis include *cumulative* viewsheds (i.e. joint coverage areas covered by two or more towers), *subtractive* viewsheds (areas covered by one set of towers but not by another set) and *elevated* viewsheds (for targets above the terrain, such as a tagged perching bird assumed to be 3 m above ground). Such advanced extensions are useful for planning antenna arrays, tracking systems, communication networks, viewing towers and other applications in movement and spatial ecology (and beyond), underscoring the relevance of accessible LOS and viewshed tools.

While the features desired from viewshed tools may vary among possible applications, adding these advanced viewshed capacities (i.e. cumulative, subtractive and elevated) to the basics of simple point-to-point LOS or single tower viewshed is likely to broaden the range of applications for most users. Nevertheless, available tools (table 1 for a non-exhaustive overview) are limited either in their functionality (explicitly, lacking the ability of the above-mentioned advanced viewshed analyses) or in their accessibility for users. This methodological gap is hampering the full assimilation of these analyses into ecological and environmental studies performed by non-specialists. Several free Web tools offer only basic LOS and viewshed (tool entries nos. 1–3 in table 1). For instance, Google Earth only allows viewshed calculation from a single tower to objects at ground level within 10 km. Some tools (e.g. tool no. 4) bind users to a predefined DEM resolution, do not account for Earth curvature nor do they allow advanced calculations (e.g. subtractive viewshed). Lack of ability to analyze aquatic systems is yet another limitation. Other tools offer better flexibility and functionalities but require paid registration that can be substantial (e.g. tools nos. 5 and 6) or are accessible only to geospatial experienced users (tools nos. 5–7). Setting up LOS-based tracking systems (or similar) without consideration of the interactive viewshed of towers can result in poor performance, gaps of insufficient coverage within the study site, or loss of data and budgets when towers have to be repositioned to improve performance. A calculation tool for LOS and viewshed that is user-friendly, free (thus more accessible) and open-source (thus allowing adjustments and modifications for the qualified users) may provide a useful handle for many scholars and address the current methodological gap. Such a tool can facilitate ideal tower deployment and optimal use of available ones to ensure sufficient coverage.

**Table 1.** An overview of some of the available viewshed tools.

	tool name	website	availability	available functionalities
1	scadacore	http://scadacore.com/tools/rf-path/rf-line-of-sight/	free Web tool	LOS only, flat Earth only
2	Solwise	https://www.solwise.co.uk/wireless-elevationtool.html	free Web tool	LOS only, flat Earth only
3	Google Earth	https://earth.google.com/web/	free software	LOS and a simple viewshed only (i.e. no cumulative, subtractive or elevated); limited to a 10 km range
4	Heywhatsthat	http://www.heywhatsthat.com/	commercial Web tool with a limited tier	LOS with landmarks identification along it; cumulative viewshed additional horizon panorama view
5	Global mapper	https://www.bluemarblegeo.com/global-mapper/	commercial software	5–7 offer sophisticated capabilities but are difficult to master and use
6	ArcGIS (ESRI)	https://www.arcgis.com/	commercial software	
7	GIS (QGIS)	https://qgis.org/en/site/	free open source software	

To address this gap, here we present ViewShedR, a free, open source and easy-to-use tool to perform LOS, basic and advanced viewshed analyses. We demonstrate its functionality in the context of the design and deployment of receiver arrays for wildlife tracking. We use two case studies involving technologies based on a set of stationary towers (receivers) and animal-borne mobile transmitting tags (targets), representing common scenarios in movement ecology requiring LOS and viewshed analyses [20]. Our first case study is based on a terrestrial ATLAS high-throughput tracking system in the Harod Valley in Israel. The second case study is a hypothetical array of marine acoustic receivers placed at the Dry Tortugas in Florida, a well-studied archipelago. We first describe the functions and structure of the ViewShedR (using figures from the first case study), and then turn to the application examples and to further discussion.

## 2. Material and methods

ViewShedR calculates viewshed maps for a set of chosen towers (whose locations and heights are user-defined) within a set region. Several functions are provided, including the viewshed map of an unlimited number of towers; elevated viewsheds for any desired target height above ground; cumulative or subtractive viewshed of an array of towers. The application can also treat arrays of submerged acoustic receivers and transmitters operating in marine/aquatic environments. Here users may provide water surface altitude and bathymetric data for the viewshed analyses. The application is flexible in its ability to use DEM from any user-specified data source. It is implemented and runs within R, a programming language and environment that is widely used in ecology [21,22]. ViewShedR is designed to be intuitive for beginners while allowing flexibility for skilled users. Expert users can also adapt the source code to their specific needs. The source code and a detailed user manual can be downloaded freely from the GitHub repository https://github.com/Orrslab/ViewShedR. The ViewShedR tool itself (shiny app), is readily launched from the R console as detailed in the manual and demonstrated in a provided video tutorial (https://www.youtube.com/watch?v=De-E4qezcEM). The repository also contains the data required to reproduce the analyses presented here.

**Figure 1.**
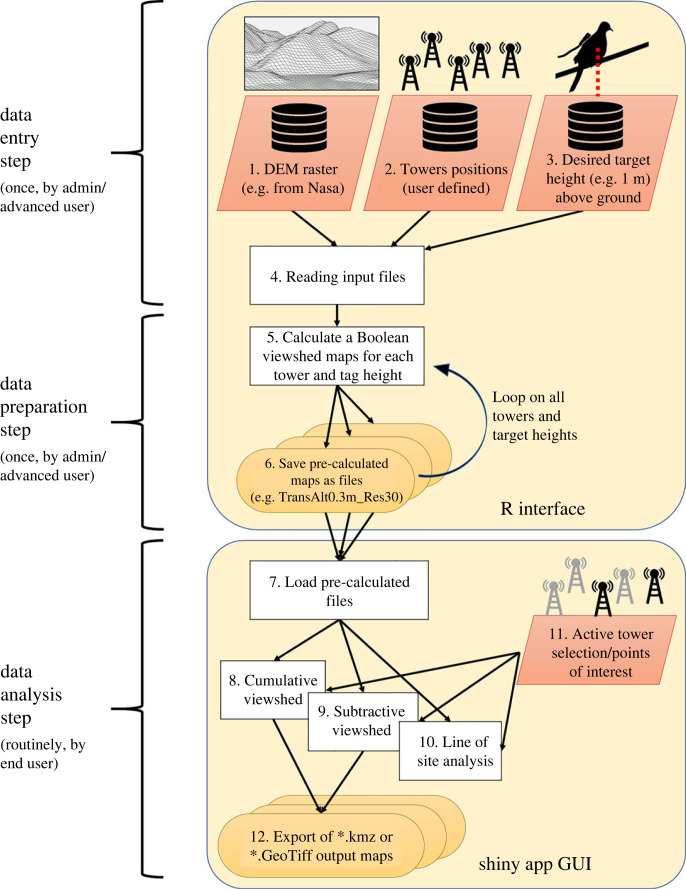
A flowchart of the ViewShedR tool and shiny app showing steps of data entry, preparation and analysis. Dark orange boxes indicate inputs to the tool and light orange ovals represent output files.

Applying and using ViewShedR includes three phases: data entry (import), preparation, and analysis (figure 1). Below we briefly describe the first two steps (which are thoroughly explained in the user manual) and then expand on the functionalities included in the graphical user interphase (GUI) of the analysis step.

*Data entry* includes defining the region of interest (aka the extent) and importing the desired DEM as a TIF file. Then the user needs to import the tower data (names, locations, height and maximal range) as a csv file. These towers will be subsequently used for LOS calculation (in the data preparation phase) and for data inquiries (within the data analysis phase). Finally, the user needs to choose the target height(s) above surface for which viewshed maps will be computed in the next step.

DEMs are freely available from various sources such as NASA Shuttle Radar Topography Mission (SRTM) [23] or the CGIAR-CSI GeoPortal [24]. Naturally, finer-scale resolution of the imported DEM will allow the ViewShedR tool better accuracy in calculation. Yet, high-resolution DEMS may slow performance, especially with a wide raster extent. We suggest that a DEM grid cell in the range of 30 × 30 m should suffice for most applications, while minimizing computational loads.

The *data preparation* step creates the basic viewshed files that will be used later to perform the analyses. This step is typically executed by a skilled user or admin, generating Boolean raster maps of the region for each tower and for each requested target height above surface (according to the input entered in the previous phase). The user can determine the maximal reception range for each tower (default is limited by the DEM extent). The maps can correspond to a flat Earth or a curved Earth model (which is recommended if longer ranges are used). These steps are time consuming, taking minutes to hours, so they are invoked via the R console. Yet, they only need to be performed once for a given tower array and region. Once obtained, the resulting viewshed maps (hereafter referred to as *pre-calculated* maps) can be stored locally and distributed to other users in the research group. This step allows any chosen analysis to be implemented easily via the GUI within seconds without any programming (i.e. also available for more novice group members).

For LOS and viewshed calculation the ViewShedR uses several functions adopted from existing packages including the ‘windfarmGA’ [25], the ‘raster’ package [26], ‘leaflet’ [27] and ‘shiny’ [28]. First, for a given DEM cell we generate a direct three-dimensional line between the cell and the tower, then we extract the elevation of all cells under that line. LOS exists if none of the cells is above line. This procedure is looped over all cells in the DEM extent, where points beyond the chosen reception distance are set to ‘false’ automatically. This generates a Boolean value for each DEM cell (for a given tower and tag height). In calculation accounting for Earth curvature, we assume that the maximal range from the tower is negligible with respect to Earth radius (set as 6731 km) and use Taylor's approximation for the square root (*x* << 1) to reduce the height of each point along the path according to its distance from the tower (see the documentation). Finally, the procedure is repeated for all available towers, resulting in a set of pre-calculated Boolean rasters that are readily available for further calculations in the analysis step. These include simple grid summation or subtraction in the cumulative or subtractive viewshed calculation, respectively. The user manual provides additional details on these procedures.

The *data analysis step* is performed in an easy-to-use interactive GUI, implemented as a shiny app. Users interact with the app using a Web browser (e.g. Chrome or Firefox) and locally available pre-calculated maps; no familiarity with the R console or the R language is required, and inquiries typically take less than 2 s. Resulting viewshed maps can be directly viewed in the browser or exported to .*kmz* or .*GeoTiff* formats for later usage.

**Figure 2.**
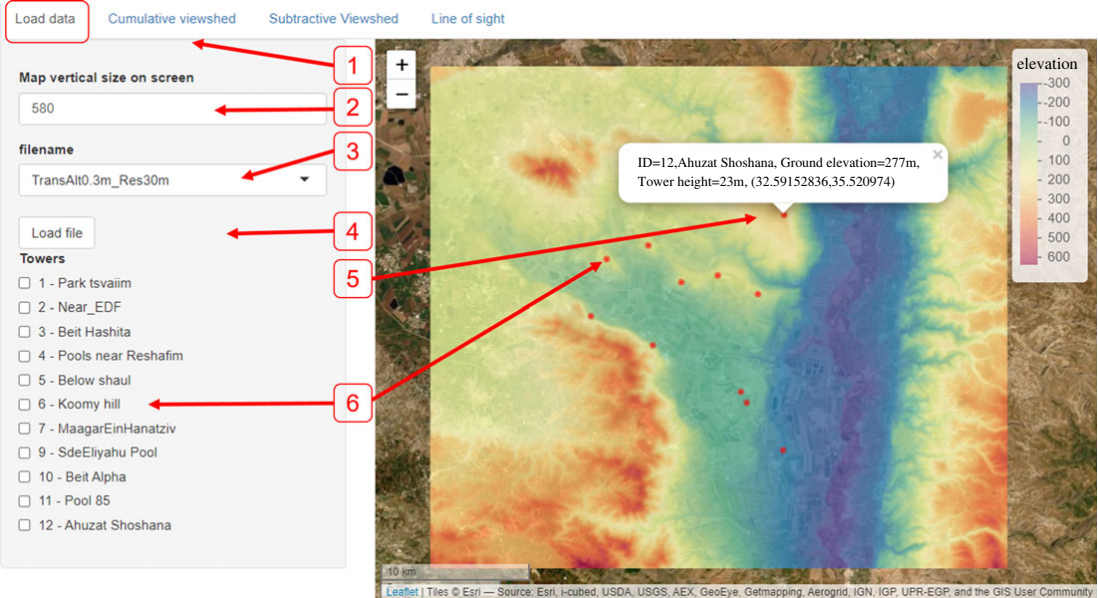
‘Load data’ tab, here the end user can choose from the available viewshed files. After choosing the ‘Load data’ tab (1), the options include: an adaptable map vertical size (in pixels, 2), to adjust to screen size; a dropdown menu (3) to choose file from available, pre-calculated files (with towers and their respective viewshed maps). These are generated by user/admin during the preceding data preparation step. ‘Load file’ button (4), to load the file chosen above. Upon clicking a tower (a red dot on the map, 5), its details will be presented (e.g. tower no. 12). The towers that are available for inquiries (i.e. for which viewshed calculation was done during data preparation step) are also presented in the checklist on the left (6). Checking a tower will highlight it on the map as a star for easier finding.

Below we expand on the GUI of the analysis step, and describe the functions it provides, each in a different tab:

*The ‘Load data’ tab* allows the user to choose and load existing files available in the designated source directory (figure 2). These files include pre-calculated viewshed layers for a specific target-height, saved by the user (or admin) at the data preparation step. We recommend that users use informative file names that encode the target height and the DEM resolution. For example, ‘TransAlt0.3m_Res30’ represents a set of viewsheds for a target 0.3 m above ground—reflecting a medium-size walking animal—with a DEM at spatial resolution of 30 m (figure 2). Once the user chooses a file and loads it, the app presents a map indicating both tower locations and terrain elevation (figure 2). This step is a prerequisite for the following optional analyses available in the other tabs.

**Figure 3.**
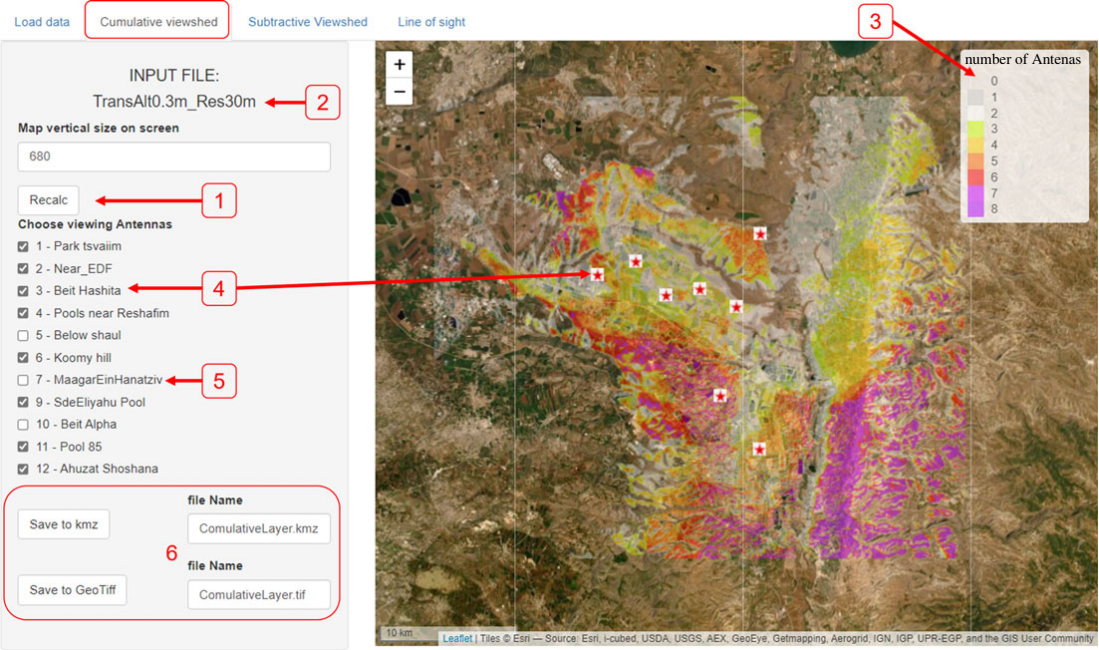
The ‘Cumulative viewshed’ tab. A map of cumulative viewshed will be presented (right) after loading data (figure 2), choosing desired towers to include (left) and hitting the ‘Recalc’ button (1). The active file name used for analysis is presented in (2). The legend (3) indicates the colour code for the number of viewing towers, with locations covered by more towers indicated in darker colours. Only ticked towers (4, marked on the map with red stars) are counted, and unticked towers (5, hidden from the map) are ignored. Saving options (6) are available at either .kmz format or .GeoTiff (the file name can be edited by the user).

*The ‘Cumulative viewshed’ tab* allows the user to choose towers (checkbox ticking) using the data in the chosen layer. The app presents the numbers of observing towers (out of the selected ones only) at every point using colour codes (figure 3).

**Figure 4.**
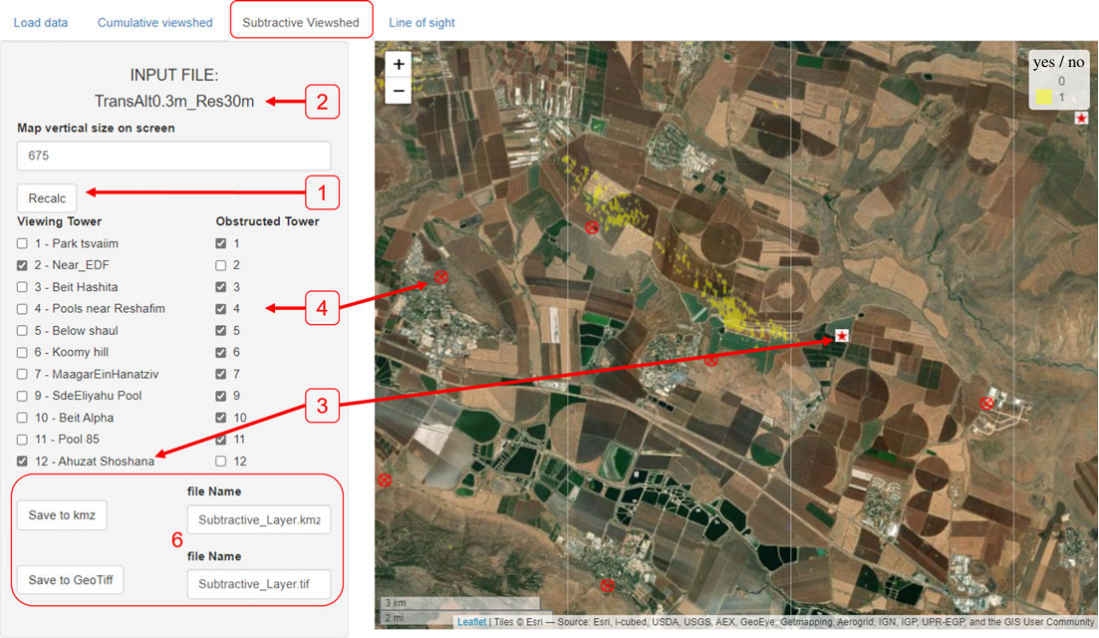
The ‘Subtractive viewshed’ tab. A map of the subtractive viewshed will be presented (right) after loading data (figure 2), choosing desired towers to include (left) and hitting the ‘Recalc’ button (1). The active file name used for analysis is presented in (2). Only ticked towers are counted, either as viewing towers (3, red stars on the map) or as obstructed towers (4, red ‘X sign’ on the map). Yellow highlighted areas represent the cells that obey the user choice of subtractive conditions (e.g. here: in sight for towers nos. 2 and 12 but obscured for all other active towers, as indicated by the checked boxes). Saving options are present at (6). This particular tower combination reflects detections of an owl-borne tag (tag no. 208) after the bird mortality event. Narrowing down search range allowed the user to recover the bird (see Results and application examples for details).

*The ‘Subtractive viewshed’ tab* is similar to the previous but allows visualization of subtractive viewsheds. The user chooses viewing towers (from which target is observable) and obstructed ones. The app presents the area visible from all the viewing towers but from none of the obstructed ones. (figure 4).

**Figure 5.**
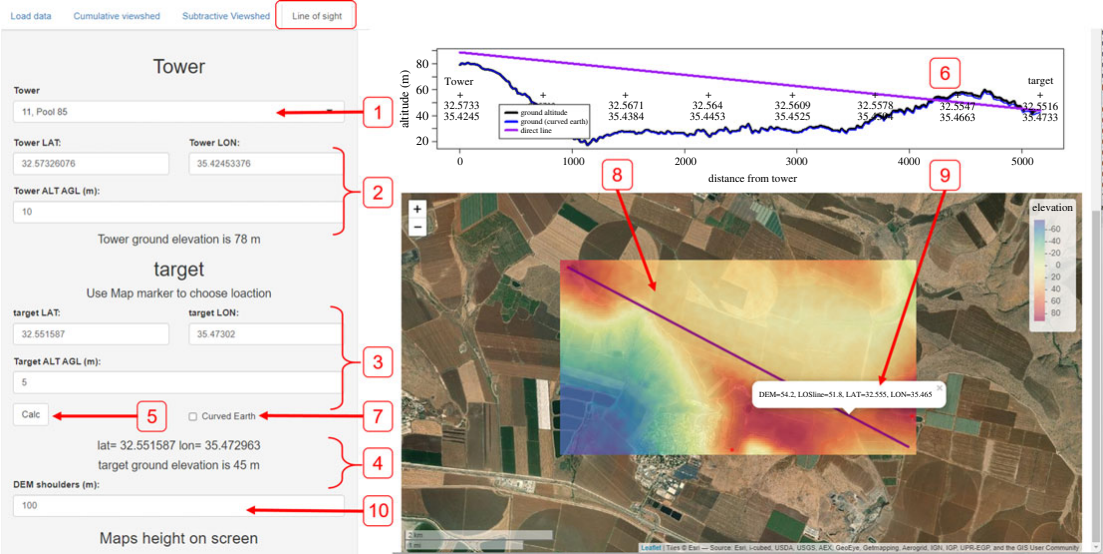
The ‘Line of sight’ (LOS) tab. The user may choose a tower from a drop-down list (1) or manually type its coordinates in Lat-Lon and height above ground (2). Similarly, the user may choose a target location (3) either by typing its coordinates or by pinpointing a location on the map (not shown here). The coordinates and ground elevation at the chosen target point will be displayed (4). Once the ‘Calc’ button (5) is pressed the LOS will be calculated and presented in the upper right inset (6). The direct line between the points (LOS) is green if clear and purple if obstructed. The DEM elevation profile along the LOS is presented with two lines, one excluding Earth curvature (black), and one accounting for it (blue). Over short distances the two often overlap, and the latter becomes default when chosen in a tick-box (7). The bottom right panel (8) presents a map line connecting the two points and a detailed DEM in the vicinity of the points. Coordinates are presented upon clicking any point along the line (9). The DEM area can be expanded by changing the DEM shoulder value (10).

*The ‘Line of sight’ tab* allows the investigation of terrain and obstacles between any two spatial locations (figure 5). The user can choose a source point, a *tower* (either from the pre-calculated list or directly by typing the coordinates and relative height) and a *target* (either by clicking the map or by inserting the coordinates and height). The app will display the terrain along the line between these two points and a detailed DEM in the vicinity (the user can define the shoulders' width). This analysis allows the investigation of the obstacles between any two points to explain the LOS results.

## 3. Results and application examples

### 3.1. A terrestrial case study—the ATLAS system

ViewShedR has been instrumental for our deployment of the ATLAS system at the Harod Valley, Israel (32.53° N, 35.43° E; [29,30]). This system is a terrestrial tracking infrastructure based on a reverse-GPS approach where tags transmit a short ID-coded radio signal (UHF) typically every 1 to 8 s [18,19,31]. Once a specific transmission is detected by at least three towers, the location of the tag can be calculated from the miniscule (nano-seconds) time-of-arrival differences among them. ATLAS systems offer equivalent spatial accuracy to those of alternative tracking methods (namely GPS telemetry) with several advantages for local tracking, including lower price level, higher energetic efficiency and real-time tracking. However, the localization depends on simultaneously detecting a transmission by several towers (at least three, ideally four or more), underscoring the importance of a LOS to each of the towers. Thus, a comprehensive planning of the distribution of the towers is essential.

The cumulative and subtractive functions facilitate comparison among alternative tower sites, clearly highlighting their respective coverage, the overall predicted coverage of the system with different configurations (figure 3), and the areas that will be covered by one but not by the other (figure 4). Before developing the ViewShedR tool, each viewshed analysis preceding a tower deployment required a substantial effort (often hours). With this tool, all maps required for choosing among alternatives became available within minutes, saving both expense and time.

Similarly, choosing optimal locations for animal trapping (for tagging) in places with good coverage routinely benefits from the cumulative tool, reflecting configuration of currently active towers (e.g. when some towers are down for maintenance, figure 3). The elevated viewshed calculations also facilitate trapping site selection by allowing us to match sites to the typical altitude of the focal species (e.g. barn owls that typically perch at 5–10 m above ground may be trapped in different locations than ground dwelling lapwings, whose height is 30 cm). Cumulative viewsheds are also helpful for identifying appropriate sites for the positioning of system beacons, special stationary transmitters that are required for tower synchronization and the operation of the ATLAS system (should be placed in sites of maximal coverage).

In addition to the tower/transmitter deployment context, ViewShedR has proven very useful in the recovery of lost transmitters. Occasionally, transmitters are being detected by one or two towers only, thus failing to obtain a position (as noted above, at least three towers are required for localization in ATLAS and similar systems). This scenario is particularly relevant in cases of mortality, in which a tag-carrying bird often falls to the ground, leading to poor reception. Yet, recovering these tags is of high biological interest for determining cause of death, and occasionally also for tag reuse. Tags can be directly detected by an observer with a hand-held Yagi antenna and a receiver. Yet, the effective reception distance for this application is limited to tens of meters and thus covering large areas is unfeasible. The subtractive analysis has been particularly effective in these cases: by considering operational towers with effective detections (viewing) and those that are currently active but obstructed to the focal transmitter, we were able to substantially reduce the uncertainty regarding the whereabouts of the tag, narrowing down the search area and facilitating tag finding. Once a small ‘suspected’ search areas has been identified (e.g. a few hectares), manual ground search becomes feasible (potentially informed also by last known location), permitting tag recovery. No partially detected tags were retrieved prior to the use of the subtractive viewshed (despite some attempted searches), and at least seven tags were retrieved with subtractive viewshed assistance. For instance, Tag no. 208 was attached to a barn owl on 1 July 2020 but on 25 August the tag could no longer be localized by ATLAS; it was only infrequently detected by two towers (2 and 12). We calculated a subtractive viewshed of these specific towers with other towers being active but obstructed (figure 4), and then successfully searched on the ground (for a couple of hours) in the highlighted areas. We retrieved the tag and identified the likely cause of death (car hit).

### 3.2. An aquatic case study—acoustic arrays

**Figure 6.**
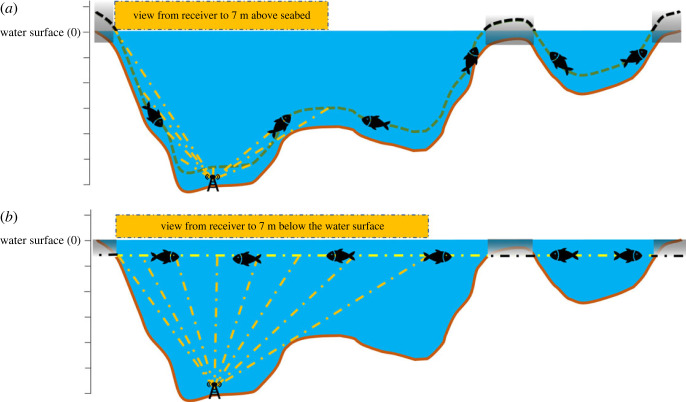
A schematic representation of LOS in an aquatic system. When used in aquatic context the water level must be determined (here, set at 0) the target height (fish icon) can be interpreted as metres above the seabed (top panel) or as metres below water surface (bottom panel). Greyed-out areas indicate areas where the LOS calculation for a certain depth above see floor is invalid (These are also marked by ViewShedR as black patches during any visualization; figure 7). The horizontal orange bars indicate the area within LOS of the receiver (antenna icon) in the two cases.

**Figure 7.**
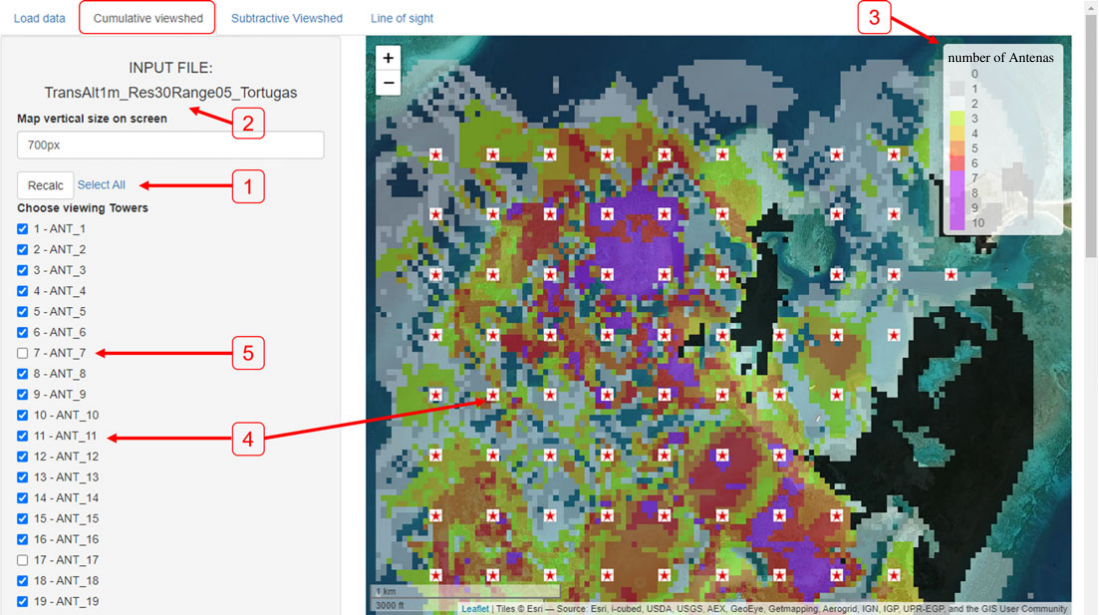
The ‘Cumulative viewshed’ tab applied to an hypothetical array located in the Dry Tortugas National Park area. The location of the receivers (the marine equivalent of terrestrial towers) was set as grid, spaced 300 m apart, and 1 m above the seabed. The active file name used for analysis is presented in (2). Choosing desired receivers to include (4) and hitting the ‘Recalc’ button (1) generates a coloured layer displaying the number of viewing receivers at each point with a corresponding legend (3). Only ticked receivers (4, marked on the map with red stars) are counted and unticked receivers (5, hidden from the map) are ignored. Note that islet shadows preventing LOS calculation are indicated by black shadows.

ViewShedR is also applicable for aquatic and marine systems where receivers (instead of towers) and tags are submerged underwater and bathymetry landscape determines the viewshed (figure 6 for a schematic example). To demonstrate this, we applied these analyses on a hypothetical grid array, placed in the ‘Dry Tortugas’ archipelago, where several acoustic telemetry ecological studies have been done [32–34]. The bathymetric data was obtained from NOAA [35]. Because locations of a real array were not available, we created a grid of 90 receivers, placed 1 m above seabed (following [32]) and 300 m apart of each other. Receivers have an effective distance of 500 m. The cumulative analysis results demonstrate how underwater ridges and islets may restrict coverage by multiple receivers (figure 7). While these results are not intended to provide specific guidance because they merely reflect a hypothetical array over real bathymetry, they do demonstrate the potential value of ViewShedR for optimizing submerged aquatic arrays that are widespread in ecological studies [17].

Note that in contrast to terrestrial applications, aquatic systems require the elevation of the water surface (typically approx. 0 for oceans but not for lakes; figure 6) and the target (tag) height can be set either as constant height above the seabed (equivalent to height above ground in terrestrial system) or at a constant depth below water surface (e.g. for pelagic species). Working on aquatic system might render some areas as invalid for LOS calculation (e.g. calculating the LOS for 5 m above seabed for 3 m deep area)—these areas are detected and marked by ViewShedR (black patches in figure 7).

## 4. Discussion

ViewShedR is a new free open-source application for LOS and viewshed analyses applicable to both terrestrial and aquatic environments. It offers diverse functionalities with advanced viewshed calculations (namely elevated, cumulative and subtractive) and consideration of Earth curvature. These features are usually found only in products that require high level of expertise to operate and/or high licensing costs. The tool is user friendly, and after a simple data preparation it allows even novice users (with no programming capabilities) to produce meaningful inquiries with a simple graphical user interface. The implementation of the tool within the R language that is widely used by ecologists and life scientists, further facilitates the assimilation of ViewShedR by non-experts. The open-source nature of the tool, in contrast, allows expert users to add features to support a variety of applications and settings, including academic research, teaching, landscape planning and commercial use. Below we briefly discuss some of the current limitations of ViewShedR, as well possible improvements and future directions of viewshed analyses and their general applications in the context of tracking arrays and beyond.

Several limitations should be considered while using the ViewShedR. First, calculation accuracy depends on the quality of input parameters, especially of the chosen DEM. Whereas various DEMs with a resolution of 1 arc-second (approx. 30 m) can be found on the Web, they may vary in their qualities. DEMs typically do not include land cover (buildings and trees), potentially introducing errors and inconsistencies, both in the tower height as well as in the LOS itself. This is particularly important in urban areas where buildings block LOS and signals but might vary in their inclusion in the DEM. Second, currently the ViewShedR addresses only the first-order concern regarding an LOS without additional considerations, but users utilizing the application in the context of radio signal communication (as done here) should note that radio signals may be affected by atmospheric refraction and reflections. Complex interactions among obstructions, absorption and dispersion of the propagating signals may result in deviation from predictions based on LOS. These phenomena may occasionally enable reception among points with no clear LOS in some frequencies, or result in failed localization despite LOS existence to towers [36]. Lastly, while offering a simple tool for assessing viewshed in diverse ways, the ViewShedR tool still requires manual investigation of possible sites and combinations, and has no automated algorithm to suggest optimal array deployment.

To address these limitations, we envision several future improvements of ViewShedR. Some are quite simple while others appear to be more challenging. First, accounting for partial attenuation by land cover (e.g. vegetation and buildings) can be incorporated into the tool by adding Boolean raster maps of specific vegetation types and adding vegetation-specific parameters (e.g. obtained by drones mapping or Lidar; e.g. [37]). With this envisioned feature, a user would be able to add a map that indicates, for example, forested areas, and set a lower distance-to-receiver threshold for targets in the forest, reflecting the expected attenuation of radio signals. Second, algorithms for automatically optimizing receiver placement would add highly valuable functionality to ViewShedR, but are probably more challenging to implement. Planning an optimal array design (e.g. ideal coverage with N towers on a given plot; [38]) will also require users to set constraints on tower height (terrestrial) or receiver depth (aquatic), and minimal distances among them. This potential next step can be particularly useful in systems with a large number of potential locations for towers (or receivers). However, in many cases (as in the above-mentioned ATLAS example) selection is limited by site availability to a discrete number of possibilities. One may only choose among a few options with suitable infrastructure (e.g. existing communication towers, or power sources in wilderness areas). In these latter systems, the resulting optimization problem is combinatorial (which N placements to use of the possible M). This problem is presumably simpler than the former continuous optimization problem, which might be computationally hard. Finally, other improvements can include improved graphics and user interface that were not achievable in this initial development, as well as improved computational efficiency.

## 5. Concluding remarks

Radio and acoustic telemetry are two approaches for animal tracking with growing significance and use [17,19]. Both of these ecological applications can benefit from LOS and viewshed tools in several ways, including better and more homogeneous cover, and simpler and more efficient selection of sites for receiver deployments. Interestingly, some derived viewshed capacities can further improve tracking efforts, as for the case of subtractive viewshed allowing to pinpoint areas for manual search of lost tags (figure 4), and facilitating tag reuse and identification of mortality causes [29].

Other fields that use this kind of analysis regularly are archaeology [3], where, for example, the meaning of significant structures can be deduced according to their visibility [2,5]; in landscape architecture viewshed is used for forest management and renewable energy planning [7,9]. ViewShedR should be useful for scholars in these fields, especially for those who already use the R language and require a LOS analysis tool without the use of external, sophisticated geographical tools. Many of these fields, and ecology in particular, are constantly being revolutionized by new technologies that improve our ability to describe and investigate the nature around us [11,12,14]. Movement ecology using animal tracking systems may benefit from the support of advance and accessible LOS and viewshed analysis. Thus ViewShedR may contribute to new insights that facilitate conservation and wildlife management. The authors have used the tool to optimize an existing array of terrestrial radio receivers and for a few specialized applications associated with this array, and other colleagues have been using it for the planning phase of similar arrays. While we demonstrate its potential also for planning and improving underwater ultrasonic arrays, to the best of our knowledge these applications have not been done yet, awaiting future users to test them in practice.

## Data Availability

Data and relevant source code for this research work and presented tool, as well as a detailed user manual are all stored in GitHub: https://github.com/Orrslab/ViewShedR, and have been archived within the Zenodo repository: https://zenodo.org/badge/latestdoi/360513078, http://dx.doi.org/10.5281/zenodo.8002042 [39]. In addition, a tutorial video is available at https://www.youtube.com/watch?v=De-E4qezcEM.
